# Incidental context information increases recollection

**DOI:** 10.1101/lm.042622.116

**Published:** 2017-03

**Authors:** Kamar E. Ameen-Ali, Liam J. Norman, Madeline J. Eacott, Alexander Easton

**Affiliations:** 1 Department of Psychology, University of Durham, Durham, DH1 3LE, United Kingdom

## Abstract

The current study describes a receiver-operating characteristic (ROC) task for human participants based on the spontaneous recognition memory paradigms typically used with rodents. Recollection was significantly higher when an object was in the same location and background as at encoding, a combination used to assess episodic-like memory in animals, but not when only one of these task-irrelevant cues was present. The results show that incidentally encoded cue information can determine the degree of recollection, and opens up the possibility of assessing recollection across species in a single experimental paradigm, allowing better understanding of the cognitive and biological mechanisms at play.

Recognition memory is the ability to identify that something has been previously encountered. In humans, episodic memory is associated with a subjective experience of remembering (Tulving and Markowitsch 1998). As the subjective experience of nonhuman animals cannot be assessed, the term “episodic-like” memory is often used when memory for an event containing information about objects in specific combinations of location and spatial/temporal context is demonstrated (what, where, and when) (Clayton and Dickinson 1998); (what–where–which occasion) (Eacott and Norman 2004).

Human tests of episodic memory have been used to provide insight into the distinct, independent processes of familiarity (a feeling of having previously encountered the object/event without any additional information) and recollection (the bringing to mind information from the encoded event which is not presented at test). There is a general consensus that familiarity can be considered a continuous variable, but there remains debate regarding whether recollection is a continuous rather than a noncontinuous threshold variable (e.g., Wixted 2007; Yonelinas et al. 2010).

The analysis of receiver-operating characteristics (ROCs) has been applied to recognition memory, where participants typically provide confidence ratings alongside recognition judgements. Performance is plotted as an ROC curve with hit rate (HR—the probability of a stimulus being correctly identified as “old”) against false alarm rate (FAR—the probability of a stimulus being misidentified as “old”). The dual-process signal detection model (DPSD) (Yonelinas 1994; Yonelinas et al. 1998, 2002; Fortin et al. 2004; Aggleton et al. 2005; Sauvage et al. 2008) states that symmetrical curvilinear functions represent familiarity-based responses—a continuous signal detection process whereby recognition accuracy depends upon the strength of familiarity. Asymmetrical curvilinear functions result from both a continuous curvilinear function (familiarity), and a noncontinuous linear function that may represent the threshold nature of recollection (e.g., Yonelinas 1994; Morris and Rugg 2004). The asymmetry from including a recollection-based linear function is the result of a high confidence hit rate with no effect of false alarm rate (Yonelinas 1994). Theoretical-based models can be fitted to the data using a regression method or a maximum likelihood estimates method, which allow for parameter estimates of memory processes to be assessed (Yonelinas and Parks 2007). As the degrees of recollection and familiarity can be quantified, only the need for introspective assessment of the confidence of one's memory, rather than a categorical description of it (e.g., “remember” versus “know”), is required. This allows human and nonhuman animal memory to be understood in a more similar manner.

The current study used ROC analysis to obtain quantifiable measures of recollection and familiarity in a task based on recognition memory paradigms typically used with rodents, derived from the “what–where–which occasion” episodic memory descriptor (memory for an object, its location, and background context; Eacott and Norman 2004). Participants completed a computer-based memory task, making old/new judgements about objects. The old objects could be shown at test in the same configuration of object, location, and context (OLC) as previously seen, same object and location but different context (OL), same object and context but different location (OC) or the object in different location and context (object recognition: OR). As the OLC condition, by definition, is akin to the what–where–which occasion descriptor used to infer episodic-like memory in rodents, it was hypothesized that significantly greater recollection would be elicited in this condition relative to the other recognition conditions if this process underlies episodic memories.

A single testing block consisted of 10 encoding-retrieval phases (see Supplemental Materials for more task information). An encoding phase began with four objects presented sequentially (Fig. 1). Each of the four objects was presented in a unique combination of location (left/right) and context (context A/context B), such that each context and location was experienced an equal number of times in each encoding phase with no combination repeated. An object was never repeated within the same block of encoding trials (i.e., the block of 10 encoding-retrieval phases). Participants were instructed to move their eyes to the object when it appeared, and then back to the fixation cross when the object disappeared. A retrieval phase followed wherein four objects were shown sequentially, each of these constituting a single retrieval event (Fig. 1). These objects could be assigned to any one of the “old” object conditions (OR, OL, OC, or OLC) or the “new” object condition. Participants made two responses after viewing each object in the retrieval phase: first whether the object was old (i.e., it had appeared in the previous encoding phase) or new (i.e., it did not appear in the encoding phase), and secondly, participants rated how confident they were with their judgement (1 = guessing; 2 = not very confident; 3 = quite confident; 4 = very confident). The old/new judgement was to be made entirely on the object identity, and context and location were not relevant to this judgement. Each of the objects in the retrieval phase could either be new or old, relative to the immediately preceding encoding phase. If the object was new, it was presented in a random combination of context and location. If it was old, the context and location depended on the condition for that trial (OR, OL, OC, or OLC).

**Figure 1.**
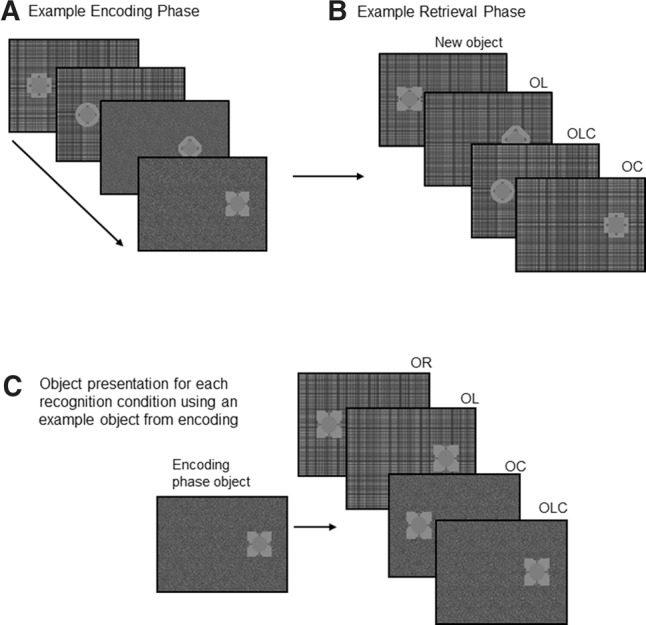
Experimental procedure. A single testing block consisted of encoding and retrieval phases. Example of object presentation for an encoding phase (*A*). Four objects are sequentially presented for 2 sec each, separated by a fixation cross presented for 2 sec (not shown in the image). The objects are presented in a unique combination of location and context such that in each encoding phase the *left* and *right* locations and contexts *A* and *B* would be experienced an equal number of times, but presented in a randomly selected order. Example of object presentation for a retrieval phase (*B*). Four objects (preceded by a 2-sec fixation cross) are sequentially presented for 2 sec each, that may be old or new relative to the objects that appeared in the encoding phase, with each of these retrieval object presentations constituting a single trial. New objects would be presented in a random combination of location and context. Participants were instructed to respond whether the presented object was old or new, relative to the immediately preceding encoding phase, and their confidence rating. The next object was not presented until these responses had been collected. Example object presentation for a retrieval phase (*C*) that illustrates the possible location and context configurations for a single object from encoding, depending on the old object condition in which it may feature. For old objects, the potential locations and contexts are determined by condition; object recognition (OR) objects are presented in a novel location and context relative to their appearance in the encoding event; object–location (OL) objects are presented in the same location but novel context; object–context (OC) objects are presented in the same context but novel location; and object–location–context (OLC) objects are presented in the same location and same context relative to their appearance in the encoding event.

After four retrieval events had been completed, a tone signaled the start of the next encoding phase. Each testing block, therefore, consisted of 40 events (four events per encoding-retrieval phase pair, and 10 encoding-retrieval pairs per block). In total, participants completed 16 testing blocks over 4 d (four 10-min testing blocks per day), with each block consisting of 10 encoding-retrieval phases (a total of 640 retrieval events).

Data from all 16 blocks were analyzed collectively for each participant and transformed from response frequencies to accumulated probabilities that represent points on the ROC curve. FAR probabilities were derived from the new object condition, and separate HR probabilities were derived from each of the four old object conditions. The ROC function was fitted to the data using a method of least-squares (Fig. 2). The best fitting ROC curve for each set of points was determined using the following dual-process equations (Yonelinas et al. 1998):

$$\begin{array}{rl} \mathrm{HR} & =R+(1-R)F_{\mathrm{old}}\\ F_{\mathrm{old}} & =\Phi \left(\frac{d^{\prime }}{2-c_{i}}\right)\\ \mathrm{FAR} & =F_{\mathrm{new}}\\ F_{\mathrm{new}} & =\Phi \left(\frac{-d^{\prime }}{2-c_{i}}\right). \end{array}$$

**Figure 2.**
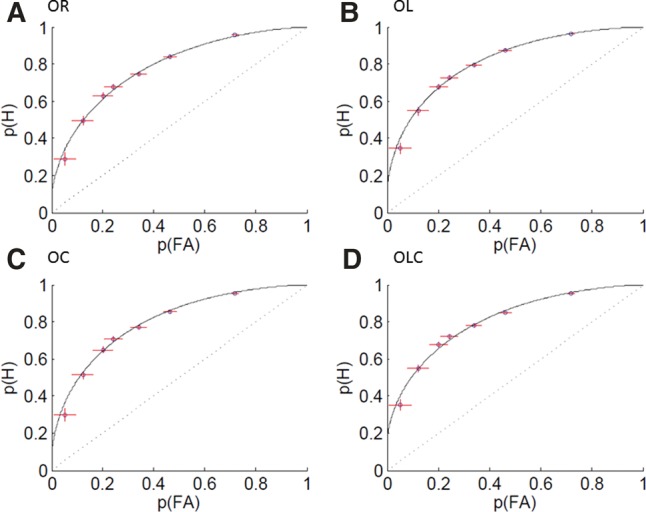
ROC curves with hit rate (HR) plotted against false alarm rate (FAR) for all subjects. Standard object recognition (OR) memory (*A*). Object–location (OL) memory (*B*). Object–context (OC) memory (*C*). Object–location–context (OLC) memory (*D*). The horizontal SEM bars show the variance for FAR, and the vertical SEM bars show the variance for HR.

Ф is the cumulative normal distribution; *c* is the calculated criterion values reflecting an individual's response bias; and *i* is the index for the different criterion levels.

For each ROC curve, the derived parameters of *d*′ (a measure given by the amount of separation, in standard deviation units, between the distribution in memory strength elicited by novel items, and the distribution in memory strength elicited by familiar items) were taken as a quantifiable measure of familiarity as it is thought to reflect a signal detection process. Equally, the *R* probability (a measure of the threshold process of recollection given by the probability of there being a recollective experience) was taken as a quantifiable estimate of recollection, and compared across conditions rather than being taken as an absolute measure, as *R* probability is likely to be an underestimate of true levels of recollection. *d*′ was found to vary with task conditions (OR = 1.06; OL = 1.21; OC = 1.16; OLC = 1.10; *F*_(3,63)_ = 5.152, *P* = 0.003; Fig. 3A,B), with the significant effect lying between the OR and OL memory conditions only (*P* = 0.015), with *d*′ being higher in the OL condition. The level of *R* probability was also found to vary with task conditions (OR = 0.10; OL = 0.12; OC = 0.09; OLC = 0.17; *F*_(3,63)_ = 5.075, *P* = 0.003). *R* probability in the OLC condition was found to be significantly greater than any of the other recognition conditions (OR and OLC: *t*_(21)_ = 3.864, *P* = 0.001; OL and OLC: *t*_(21)_ = 2.238, *P* = 0.036; OC and OLC: *t*_(21)_ = 3.472, *P* = 0.002), whereas none of the other recognition conditions were significantly different from each other (all *P* > 0.250).

**Figure 3.**
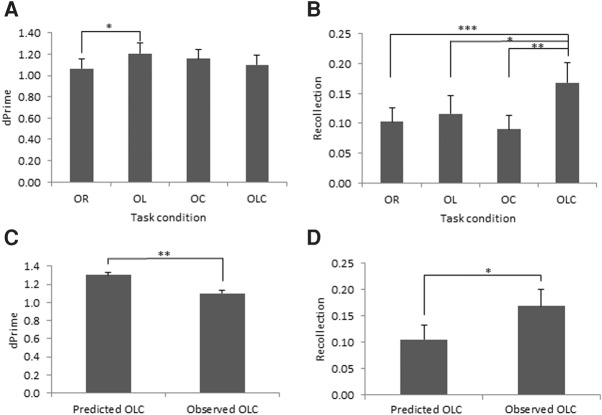
ROC analyses. Mean *d*′ estimates for each recognition condition (*A*): Object recognition (OR); object–location (OL); object–context (OC); object–location–context (OLC). Mean recollection probability estimates for each recognition condition (*B*). Predicted and observed *d*′ (*C*) for the object–location–context (OLC) recognition memory condition. Predicted and observed *R* probability values (*D*) for the OLC condition. Vertical bars show the standard error of the mean. (*)*P* < 0.05; (**)*P* < 0.01; (***)*P* < 0.001.

The area under the ROC curve (AUC) for each task condition was used as a single measure of performance (and thus an index of task difficulty), ranging from chance (0.50) to perfect performance (1.00). Here, ROC performance is reduced to a single scalar value measured as a portion of the area of the unit square. Generally, this means that increases in performance will result in a greater measured area (Fawcett 2006). This measure does not discriminate between different memory processes. The AUC was calculated using the trapezoidal rule for approximating the definite integral. The level of AUC varied with task condition (OR = 0.78; OL = 0.81; OC = 0.80; OLC = 0.81; AUC: *F*_(3,63)_ = 8.082, *P* = <0.001), with the OR condition significantly more difficult than the OL (*P* = 0.002) and OLC (*P* = 0.010) conditions. Crucially, the OLC condition was not significantly less difficult than the OL or OC conditions (both *P* > 0.3); therefore, the results cannot be attributed purely to differences in task difficulty.

To assess whether the combination of location and context in the OLC condition elicited a degree of recollection that is greater than that predicted by the summation of the separate degrees of recollection associated with location and context alone, the observed *R* probability in the OLC condition was compared with a hypothetical expected value predicted by the combined probability of the location and context components (see Supplemental Material for calculations). The observed *R* probability value (0.17) (Fig. 3) was significantly greater than that predicted by the summation of the OL and OC probability values (0.11; *t*_(21)_ = 2.642, *P* = 0.015). Similarly, the observed *d*′ for the OLC condition (1.10) was compared with a hypothetical expected *d*′ (1.30; Fig. 3), and was found to be significantly lower than the hypothetical expected value (*t*_(21)_ = 3.133, *P* = 0.005). Recollection in the OLC condition appears to be distinct from the simple summation of additional cue information.

The results show that recollection was greater for objects presented in the same combination of location and background context as seen at encoding. Importantly, this pattern of results was not found for the measure of familiarity, indicating that only the OLC condition leads to participants being more likely to use recollection to successfully recognize the object. This interpretation relies on the assumption that *R* and *d*′ are measures of recollection and familiarity, respectively. It should be noted that both recollection and familiarity may contribute toward performance when it is highly confident, as the ROC method leaves open the possibility that familiarity underpins highly confident responses, either instead of, or alongside, recollection. Nonetheless, the difference observed in the experiment shows clearly that whatever the underlying mechanism, the OLC condition produces a step-wise change relative to other conditions. For the reasons outlined below we believe the result is best understood in terms of an increase in levels of recollection, rather than an increase in confidence of a single familiarity process, but acknowledge that the ROC method can only ever be one line of evidence in support of this interpretation.

The results are unlikely to support the notion of a dependent relationship between recollection and familiarity. Although recollection significantly increases for OLC relative to OL/OC, the decrease in familiarity for OLC relative to OL/OC conditions is not significant, which we might expect to see whether the relationship was dependent, or whether recollection in this instance reflected high confidence familiarity. In addition, the increase in recollection from OL/OC to OLC cannot be attributed to performance change between OL/OC and OLC conditions, because although there is a slight performance increase between OC and OLC, this is not significant and therefore not sufficient to account for the significant increase in recollection for OLC.

Participants may have attended to the contextual and location information despite only being instructed to remember the object. However, levels of recollection were not significantly different in the OL and OC conditions relative to the OR condition, where only the object was the same as at encoding, suggesting that the location and context information continue to be incidentally encoded.

Each piece of information may act as an independent cue for recollection. These cues are incidental—participants may be aware of them changing, but are not made aware that they are relevant when responding whether an object is old or new. During encoding, participants do not know which condition the objects will be assigned. Therefore, even if they explicitly encode the location and context information for the encoded objects, this should benefit performance across all conditions as they will not be able to encode cues differently across conditions.

The provision of two features that match encoding (location and context) might merely provide cues to recall, that summate to produce the observed increase in recollection in the OLC condition. However, the amount of recollection in the OLC condition was found to be significantly greater than the hypothetical expected value found through combining the recollection probabilities associated with the location and context components alone. The correct combination of object, location, and context elicits a degree of recollection-based memory that is distinct from the summation of those individual components. This strongly suggests that the increased recollection in the OLC condition is not merely a result of summative effects arising from presentation of additional cues for recall.

Evidence from source memory accuracy (Harlow and Donaldson 2013) shows that recollection is a threshold event. Using this framework, it is possible that in our experiment there may be a threshold level of object, location, and context information required to trigger recollection. In our OR, OL, and OC conditions the information may sit on one side of this threshold with the OLC condition sitting on the other side, producing the observed step change in recollection in the OLC task. Alternatively, the results might be a result of additive effects of additional cues, but that these additive effects are supra-linear with all three cues (OLC) providing much more recollection than expected from the difference between one cue (OR) and two cue (OL or OC) conditions. However, both these interpretations rely on each individual cue providing additional information for recall of the encoded memory. Rather than recollection being for the associations between the individual components, it may instead be recollection for the “scene” or “snapshot” at the time of the event, and that this “scene” is much more than the sum of its parts (Gaffan 1992, 1994). This interpretation is in line with studies of OLC memory in animals, showing that this type of memory is quantitatively different from memory for individual or pairs of components (Gaffan 1994; Eacott and Norman 2004; Eacott and Gaffan 2005; Norman and Eacott 2005; Easton et al. 2011).

In summary, manipulating incidental information at retrieval produces a step-wise change in the ROC curve only when all of object, location, and context are congruent with their presentation at encoding. This change does not occur in a linear manner with the addition of recall cues, but instead represents a shift only in the OLC condition. Making the assumption that *R* is a measure of recollection (rather than a mixture of recollection and familiarity), we believe this reflects an increased likelihood to recollect information in this condition, above all other conditions. In combining our understanding of episodic-like memory (object–location–context) in animals with the ROC approach in humans we provide a novel insight into the way in which this type of information is remembered. Only through such linking of animal and human work can we produce improved translation of recognition memory research from animals to humans.

## Supplementary Material

Supplemental Material
