# Supplementary material for: Incidental context information increases recollection
Source: Learn Mem. 2017 Mar;24(3):136–9. doi: 10.1101/lm.042622.116 (PMC5311382; doi:10.1101/lm.042622.116)
Supplement: Supplemental Material [file supp_24_3_136__index.html]

Supplemental Material 

# Incidental context information increases recollection

## Supplemental Material

- Supplemental\_Material.docx
