# Supplementary material for: Incidental context information increases recollection
Source: Learn Mem. 2017 Mar;24(3):136–9. doi: 10.1101/lm.042622.116 (PMC5311382; doi:10.1101/lm.042622.116)
Supplement: Supplemental Material [file supp_24.3.136_Supplemental_Material.docx]

Supplementary Materials

*Methods*

Twenty-two participants were recruited to the study (the total number that responded to the study advertisement and completed all testing sessions), and thus the stopping rule was merely the total number recruited within a given time-frame. All participants were naïve to the purpose of the study, and informed consent was acquired before testing took place. Participants were undergraduate and postgraduate students from the Psychology Department, Durham University, and were compensated for their time with either course credit of financial compensation. This study was covered by approval from the Durham University Psychology Department Ethics Committee.

A custom set of 64 2D virtual objects were generated using Matlab (MathWorks). Each object was a unique permutation of three components (object back surface, front surface and peripheral feature), of which there were four variations of each. Two background contexts were used which were also generated in Matlab. Context A was a chequered pattern and context B was a granulated surface pattern; both defined by grey-scale variations in luminance. Stimuli were presented on a CRT monitor using a Cambridge Research Systems (Rochester, England) ViSaGe graphics system. The monitor had a resolution of 1280x1024 pixels and ran with a refresh rate of 85Hz. The viewing distance was set at 45cm with participants resting their head on a chin rest. Each object had a width subtending 6.4° of visual angle and each object was presented 12.8° (either to the left or right) from the centre of the screen.

A single testing block consisted of 10 encoding-retrieval phases. An encoding phase began with an auditory tone lasting 1s, after which a fixation cross would appear in the centre of the screen lasting 2s. This was then followed by four objects presented sequentially for 2s each (Figure 1), and separated by periods of 2s fixation. Each of the four objects was presented in a unique combination of location (left/right) and context (context A/context B), such that each context and location were experienced an equal number of times in each encoding phase with no combination repeated. An object would never be repeated within the same block of encoding trials (i.e., the block of 10 encoding-retrieval phases). Participants were instructed to move their eyes to the object when it appeared, and then back to the fixation cross when the object disappeared. A retrieval phase followed after an auditory tone lasting 1s. In the retrieval phase four objects were shown sequentially, each of these constituting a single retrieval event (Figure 1). Again, these objects were presented for 2s each, and preceded by 2s fixation. These objects could be assigned to any one of the ‘old’ object conditions (OR, OL, OC or OLC) or the ‘new’ object condition. Participants made two responses after viewing each object in the retrieval phase: first whether the object was old (i.e., it had appeared in the previous encoding phase) or new (i.e., it did not appear in the encoding phase), and secondly, participants rated how confident they were with their judgement (1 = guessing; 2 = not very confident; 3 = quite confident; 4 = very confident). Note that the old/new judgement was to be made entirely on the object identity and that context and location were not relevant to this judgement. The next object in the retrieval phase was not presented until the participant’s responses had been recorded. Each of the objects in the retrieval phase could either be new or old, relative to the immediately preceding encoding phase. If the object was new, it was presented in a random combination of context and location. If it was old, the context and location depended on the condition for that trial (OR, OL, OC or OLC).

After four retrieval events had been completed, a tone signalled the start of the next encoding phase. Each testing block, therefore, consisted of 40 events (four events per encoding-retrieval phase pair, and 10 encoding-retrieval pairs per block). Of these events, there was an equal number (8) of events from the novel, OR, OL, OC and OLC conditions. This design required 48 unique objects for each testing block, which were determined randomly from the 64 available at the start of each block. This meant that some objects would be seen in multiple blocks but never within the same block. In total, participants completed 16 testing blocks over four days (four 10 minute testing blocks per day), with each block consisting of 10 encoding-retrieval phases (a total of 640 retrieval events). This design, therefore, yielded a total of 128 trials per experimental condition (new object condition and four recognition conditions).

In the OR condition, the objects in the retrieval phase were presented in a novel location and context relative to their appearance in the encoding phase. This is in contrast to OR trials with rodents (though based on the same principles), whereby objects are not presented in novel contexts or locations, relative to encoding in a sample phase. In such tasks, recognition is signalled by object exploration driven by novelty of the object alone, due to rodents’ innate novelty-seeking behaviours. With humans, there is no need to rely on novelty preference; as such, for the OR condition, the only familiar feature is the object. In the OL and OC recognition conditions, the objects were presented either in the same location but novel context (OL), or the same context but novel location (OC). Again, this is different to the rodent tasks in which novel configurations of object and location, or object and context, drive exploration and signal recognition. With no need to rely on novelty preference, the OL and OC conditions are designed so that familiarity is defined only by the object and location (OL), or the object and context (OC). Finally, in the OLC condition, following the same principles, the objects were presented in the same location and the same context relative to their appearance in the encoding phase rather than a novel configuration of these features, being equivalent to rodent tests of episodic memory (Eacott and Norman, 2004).

The response frequencies were tabulated at each of the eight response levels (old or new, each with four confidence levels) and converted to cumulative response probabilities by dividing the frequencies by the total number of responses in each condition. The probabilities from the highest criterion (“definitely old”) to the lowest (“definitely new”) were cumulatively added. A set of *n* categories, in this case 8, gives n-1 points on the ROC curve. There were, therefore, seven points plotted on these ROC curves. In total, four individual sets of seven ROC points were derived, each one representing either OR, OL, OC or OLC memory.

Every participant completed 16 blocks of trials, with a single block consisting of 10 encoding-retrieval phases, and each encoding-retrieval phase consisting of four encoding events and four retrieval events. The 16 testing blocks completed by each participant consisted of 160 encoding-retrieval phases in total, or 640 individual retrieval events. These 640 individual retrieval events were equally divided across the five conditions; ‘new’, OR, OL, OC or OLC, that occurred equally often (i.e., there was a total of 128 new events and 128 of each recognition condition retrieval events) that were presented randomly across each testing session. Therefore, not every retrieval phase featured a novel object.

The parameters of *d’*, c (calculated criterion values that reflect an individual’s response bias) and R probability were free to vary to provide the most suitable account of the data, with the only constraint being that 0 ≤ R ≤ 1.

*Additional analyses*

The mean sum of squared errors (SSE) between the observed and predicted data was extremely low for each condition, indicating the best fitting parameters were obtained for each condition and that the ROC curves provided a close fit to the data (OR: mean SSE = 0.003; OL: mean SSE = 0.002; OC: mean SSE = 0.003; OLC: mean SSE = 0.002).

In addition to the least-squares method, we also performed a maximum-likelihood fit using the open-source toolbox at <https://github.com/jdkoen/roc_toolbox>. The results from the two methods are similar and only those from the least-squares method are reported in the paper.

### *Predicted OLC performance*

An important question to consider is whether the correct combination of location and context in the OLC condition elicited a degree of recollection that is greater than that predicted by the summation of the separate degrees of recollection associated with location and context alone – i.e., is the greater recollection merely the result of summative effects of additional cue information? The observed R probability in the OLC condition was compared to a hypothetical expected value predicted by the combined probability of the location and context components.

First the R probability values for just the location (L_R_) and just the context (C_R_) were calculated. These values are not necessarily equal to the recollection probability values observed in the OL and OC conditions, respectively, because the recollection probability observed in the OL condition, for example, is the combined probability of that found for OR and some other unknown probability associated solely with presenting the object in the same location. The same is true for the recollection probability associated with context in the OC condition. Following the laws of adding independent probabilities, we can express this in the following way. The recollection probability observed in the OL condition (OL_R_) is equal to the recollection probability observed in the OR condition (OR_R_) plus some unknown degree of recollection probability associated with presenting an object in a familiar location (L_R_), minus the intersection of the two. The variables OL_R_ and OR_R_ are known probabilities observed from the experiment, but L_R_ is unknown and will be derived from the following formula:

*OL_R_ = OR_R_ + L_R_ – OR_R_*L_R_*

This equation can be rearranged to find L_R_:

*OL_R_ - OR_R_ = L_R_ - OR_R_*L_R_*

*OL_R_ - OR_R_ = L_R_ (1 - OR_R_)*

*(OL_R_ - OR_R_) / (1 - OR_R_) = L_R_*

*L_R_ = (OL_R_ - OR_R_) / (1 - OR_R_)*

The recollection probability observed in the OC condition (OC_R_) is equal to the recollection probability observed in the OR condition (OR_R_) plus some unknown degree of recollection probability associated with presenting an object in a familiar context (C_R_), minus the intersection of the two. The variables OC_R_ and OR_R_ are known probabilities observed from the experiment, but C_R_ is unknown and will be derived from the following formula:

*OC_R_ = OR_R_ + C_R_ – OR_R_*C_R_*

This equation can be rearranged to find C_R_:

*OC_R_ - OR_R_ = C_R_ - OR_R_*C_R_*

*OC_R_ - OR_R_ = C_R_ (1 - OR_R_)*

*(OC_R_ - OR_R_) / (1 - OR_R_) = C_R_*

*C_R_ = (OC_R_ - OR_R_) / (1 - OR_R_)*

The probability of observing recollection in the OLC condition was estimated by adding individual probabilities from the three other recognition conditions. This is equivalent to estimating the probability that at least one of the three independent events occurs. The probability of either *A*, *B*, or *C* happening is equal to the addition of the probability of *A* happening, the probability of *B* happening, and the probability of *C* happening, minus the combined probability of *A* and *B* happening, minus the combined probability of *A* and *C* happening, minus the combined probability of *B* and *C* happening, plus the combined probability of *A*, *B*, and *C* happening. The formula for calculating the probability of at least one of the three independent events occurring is the following:

*p(AuBuC) = p(A) + p(B) + p(C) - p(AnB) - p(AnC) - p(BnC) + p(AnBnC)*

We are considering the three factors that can induce recollection (OR, L, and C) as independent events, and therefore we could substitute them in to this equation to replace *A*, *B*, and *C*, to calculate the probability of at least one of these factors inducing recollection. The derived values for L_R_ and C_R_ were used to derive an expected R probability value for the OLC condition (eOLC) using the following formula:

*eOLC = OR_R_ + L_R_ + C_R_ - OR_R_*L_R_ – OR_R_*C_R_ – L_R_*C_R_ + OR_R_*L_R_*C_R_*

The observed *d’* for the OLC condition was compared to a hypothetical expected *d’* derived from the combination of the separate context and location cues. The estimated *d’* value for the OLC condition was calculated for each participant that could be compared to the observed value. This estimated *d’* value was calculated using the following formula:

*eOLC = SOR + (OL-SOR) + (OC-SOR)*
